# Weather Indices for Designing Micro-Insurance Products for Small-Holder Farmers in the Tropics

**DOI:** 10.1371/journal.pone.0038281

**Published:** 2012-06-21

**Authors:** Jacqueline Díaz Nieto, Myles Fisher, Simon Cook, Peter Läderach, Mark Lundy

**Affiliations:** 1 Catchment Science Centre, Kroto Research Institute, University of Sheffield, North Campus, Sheffield, United Kingdom; 2 Decision and Policy Analysis (DAPA), Centro Internacional de Agricultura Tropical (CIAT), Cali, Colombia; Pacific Climate Impacts Consortium, Canada

## Abstract

Agriculture is inherently risky. Drought is a particularly troublesome hazard that has a documented adverse impact on agricultural development. A long history of decision-support tools have been developed to try and help farmers or policy makers manage risk. We offer site-specific drought insurance methodology as a significant addition to this process. Drought insurance works by encapsulating the best available scientific estimate of drought probability and severity at a site within a single number- the insurance premium, which is offered by insurers to insurable parties in a transparent risk-sharing agreement. The proposed method is demonstrated in a case study for dry beans in Nicaragua.

## Introduction

Agriculture is inherently risky, a review of rural poverty identified exposure to risk as a major modifiable reason for chronic poverty, noting the widespread evidence that correlates risk with poverty [1]. Production risks include, but are not limited to climatic hazard, which of all the hazards agriculture faces is perhaps the most difficult one for agriculturalists to manage. Drought is the most serious of the natural hazards globally in terms of loss of life, accounting for 44% of reported deaths in the period 1974–2003 [2].

The mere expectation of drought is sufficient in some cases to reduce agricultural production. Nearly 80% of farmers interviewed in Ethiopia cited harvest failure caused by drought and other natural hazards as the event that caused them most concern [3]. Pandey *et al*. [4] revealed a huge drop in income for rice farmers in Orissa state in India as a result of drought. The impacts of drought extend beyond the loss of production. Sakurai and Reardon [5] include increases in local interest rates due to a rise in households seeking credit, a decline in farm labor demand, a reduction in local wages due to greater numbers seeking off-farm employment, drops in livestock prices due to distress sales of livestock and increases in food prices coinciding with low financial resources.

Additionally to the risk drought prone farmers face there is growing interest for weather insurance schemes for poor farmers to balance their risk as shown in a recent studies in Africa [6] and China [7]. Insurance schemes have been developed, for example, for east Africa [8] and Central America [9]. The reasons for the low uptake of index-based insurance schemes that is cited in the literature is the lack of understanding of the core concepts [6], and the lack of trust in the schemes and in insurance companies [10], [11]. An additional hindrance for wider uptake may be the low spatial resolution of climate data and lack of suitable crop yield data, which leads to high basis risk, which the farmers assume, and which makes the insurance unattractive for farmers. It is this aspect that we address in this study.

In this paper we introduce index rainfall insurance methodology as a tool that can help smallholder farmers manage the risk of drought. We then briefly recapitulate on a previous paper [12] where we looked at the possibility of using a weather generator to provide data to simulate crop yields used to design an indexed rainfall insurance instrument for smallholder drybean growers in Honduras. We then extend the method to determine the probabilities of damaging drought over the area in the north-central mountains of Nicaragua where drybeans are the main food crop. We finally discuss the need for insurance instruments that reduce basis risk by taking account of site specificity, crop variety and soil to design crop insurance instruments with emphasis on smallholder farmers.

### 1. Drought, Risk and Smallholder Farmers

Aside from drought, farmers face other environmental hazards such as hail, floods and frosts. In the north-central mountains of Nicaragua, where most of the drybeans are produced as a food staple by smallholder farmers, drought is by far the most common hazard and according to climate predictions it is going to get even dryer in the future [13]. Flood rains from tropical hurricanes do occur of course, but much less frequently than drought. Hail is rare and frost at altitudes lower than 1200 m does not occur at this latitude.

Drought is an especially serious problem for small-scale producers, most of whom do not have access to irrigation. For example, in Nicaragua only 8% of the land is irrigated [14], and almost none of this is in the central-north region where bean growers are located.

Droughts cause food and income insecurity through both acute effects and chronic secondary effects. Acute effects are immediate crop failure, which in extreme cases leads to hunger and even starvation. Secondary consequences of drought include increases in local rates of interest due to an increase in the number of households seeking credit and a decline in the demand for farm labor leading to a reduction in local wages due to greater numbers seeking off-farm employment. Livestock also suffer hunger and starvation leading to falling prices due to distress sales. Food prices increase coincidental with falling financial resources available to rural households as sources of income dry up [5].

The rural poor are often, indeed usually, found on lands that are marginal for one reason or another, such as low fertility soils, steep slopes and remoteness. They are especially vulnerable to drought. Large numbers of people are affected. Numerous studies have shown a strong link between risk, vulnerability and poverty [3], [15], [16], [17]. Poor households lack resources with which to absorb the shocks of natural hazards.

Even small disruptions in the flow of income can have serious implications for them, so poor farmers commonly use informal and self-insurance measures to avoid risk. As discussed in more detail below, while these measures can help survival (e.g. [18]), most studies conclude that they are not the most effective tools for risk management, since they reduce the impact of a hazard at the expense of more profitable activities [19], [20], [21]. Although any risk-management strategy has a cost, the poor often have no other options besides informal methods because insurance is rarely available to them. If the insurance is more attractive than the informal methods, our consultations with smallholders in Nicaragua suggests that they would welcome the opportunity to participate.

### 2. Risk and Insurance

#### 2.1 Strategies for coping with risk and their effects on livelihoods

Most of the modern measures to mitigate risk are not readily available in developing countries, hence farmers in these regions are obliged to adopt traditional informal risk coping mechanisms [22] (Table 1).

**Table 1:** Risk management tools.

Self insurance measures	Modern risk avoidance measures
Crop diversification	Production contracting
Maintaining financial reserves	Marketing contracting
Reliance on off-farm employment	Forward pricing
Other off-farm income generation	Futures options contracts
Selling family assets (e.g. cattle)	Leasing inputs
Avoidance of investments in expensive processes such as fertilizing(especially in high-risk years)	Invest in fertilizer, use long-term forecasts
Accumulation of stocks in good years	Acquiring crop and revenue insurance
Removal of children from education to work on farm	Custom hiring

(Source: Wenner and Arias, 2003; Skees *et al.*, 2001; Hess, 2003).

The implicit costs associated with informal strategies can be quite high [15], which many argue are a barrier to poverty alleviation and indeed reinforce poverty [21], [23]. If it were possible to accomplish the same risk reduction or risk transfer at lower cost using formal insurance, then this could increase household profits and reduce poverty. Traditional risk-coping mechanisms are also risk-averse strategies that use resources inefficiently and fail to exploit more productive investments and technologies that in the long term would result in more productive systems [14], [24]. For example, when faced with the possibility of losing an entire crop due to drought, farmers may lessen risk by minimizing investment in the crop by not applying fertilizer. They do this because making the additional investment increases their loss should the crop fail.

#### 2.2 Risk sharing through insurance is an option but has traditionally not been available to the poor

Formal insurance has provided benefits to individual consumers for centuries and in the last few years has also been suggested as a pro-poor tool for managing risk [25]. A growing number of micro-insurance products (products offered to insure items in the range of a few hundreds of dollars) are now being offered in poor countries in the areas of life, health and property insurance and in some cases, schemes for crop insurance. This growing interest in micro-insurance products as development tools is associated with the expansion of micro-credit schemes [20]. There is also a growing recognition of the mutual benefits of risk management as a tool for poverty alleviation. Micro-insurance is not only justified on the basis of humanitarian need.

Insurance can be thought of as exchanging the irregular uncertainty of large losses for regular small premium payments. A general rule of thumb seems to be that the larger the proportional loss in assets and income to the household, the fewer alternatives there are to recover from the loss [23]. Insurance is one of the few viable options for poor people to manage uncertain events that can cause large losses.

#### 2.3 Previous experience with insurance has not been good

Although we have made the case for crop insurance above, crop-insurance schemes in general in the tropics have a sorry record [26]. Several governments have developed crop insurance schemes. To date, most agricultural insurance has been either fully publicly owned or has involved large government subsidies to schemes operated by private companies. Unfortunately most of them have failed.

The main reason for failure of publicly-owned insurance schemes is because they were either multiple-peril or all-risk programs [26]. This means that virtually any cause of crop failure has been insured, which results in moral hazard where there is no incentive for the insured to use the best possible practices to avoid yield loss. Moreover, risks are widely correlated or systemic, that is a weather risk event affects many crops at the same time over an extensive geographic area [27]. Further problems are adverse selection (the insured knows more about the risk than the insurer) and the high transaction costs associated with sales, underwriting (to control adverse selection), and monitoring (to control moral hazard). A benefit of index insurance is that there is no need to underwrite each policy individually or to monitor for moral hazard, which greatly reduce transaction costs. Nevertheless, there are still sales costs, which are much proportionally much higher for the small policies sold to smallholders.

The Nicaraguan Institute for Insurance and Reinsurance (INISER) has developed a index insurance for groundnut (http://sagropecuarios.org/guide.php?p=9). A regional risk and vulnerability assessment was conducted to determine a unique insured sum, premium and indemnization per region. The INISER insurance scheme uses meteorological stations to calculate a unique basis risk per region and a non-crop specific algorithm to calculate the loss in yield due to a shortfall of precipitation. This approach may have the limitation that the basis risk is inadequately calculated in remote areas where there are few data and details of crop management available.

#### 2.4 Principles of weather insurance

Weather micro-insurance has been proposed as a viable tool to help farmers manage weather risk, which translates into crop production risk. The principles behind weather insurance have been widely discussed [26], [28], [29], [30], [31]. A review of the principles and experience of the insurance processes follows.

A number of factors govern the viability of insurance. Risk-sharing can only occur when both parties (the insurer and insured) have accurate information about a hazard and its likelihood. This has been the basis of insurance for over three centuries and Skees [32] maintains that a sound weather insurance product is transparent thus eliminating both moral hazard and adverse selection. Risk sharing must be broad enough to overcome co-variate risk (the risk that all crops insured in a scheme are affected), given that major weather events typically have broad geographic coverage. Many other factors are also important such as consumer demand, data availability, acceptably low delivery costs, capacity of local insurers, and an enabling legal and regulatory environment.

The probabilities of occurrence of adverse weather events that reduce crop yield can usually be estimated from historical weather data, provided that the available data captures the innate variability of the weather. In developing countries, this is rarely the case. Moreover, some areas are riskier than others. In an insurance scheme the probability of occurrence must be identified for specific areas and be agreed by both parties (symmetry of information).

Insurance based on weather indices is a relatively recent development, in which weather events, not yield, are the basis for determining indemnity payment. Compared to area-average indices, weather-based indices have the advantage that weather data are generally more accessible and reliable than yield data. This is especially the case in developing countries [32]. Weather-related crop insurance products succeed or fail on their ability to present accurate information about weather-related risks that are specifically associated with yield loss. The critical step is to identify the relationship between an insured weather event and consequent crop loss. There are those who argue that more generalized weather indices should be developed that can be used to protect households from the variety of losses that occur due to extreme weather events [33], [34], [35], [36], but we are not in a position to consider all these many components. We restrict ourselves to formulating approaches that might be useful to smallholders to confront the loss of one or more key staple crops due to unfavorable weather, rather than extreme weather events, which are very different.

A key attribute of weather-based index insurance is its simplicity and transparency, which makes them more attractive to global insurance markets [37]. Weather-index insurance also provides a hedge against the cause of the yield loss, rather than its cost, which is the underlying concept of insurance against yield reduction. This removes the need to estimate prices [26], [38], a critical component of many of the traditional yield-triggered insurance schemes.

## Results

### 1. Nicaragua Study Site

The main drybean-producing departments in Nicaragua are Matagalpa, Jinotega, Estelí and Nueva Segovia [41] in the north-central mountains. Most drybeans are produced on hilly to steep slopes [42].

For the baseline study, we chose San Dionisio, in Matagalpa Department, which is one of the major drybean producing areas of Nicaragua [43]. At San Dionisio drybeans and maize are generally grown at altitudes 500–800 meters on steep slopes; 67% of the area has slopes greater than 30%.

Nicaragua has a well-defined dry season from December to May and a rainy season from June to November. The rainy season is long enough to allow two successive crops to be grown known as the *primera* and *postrera*, separated by a short drought that usually occurs in July or August [44] called the *canicula*. Although the *primera* and *postrera* cropping periods are well defined, the onset of the rains is highly variable so that sowing date is of great importance to make the best use of both the *primera* and the *postrera* cropping periods.

The drybean varieties grown in Nicaragua are adapted to temperatures of between 17 and 24°C [42] and have a life cycle of 60–75 days. Farmers generally prefer small- and medium-seeded black and red types [45].

Temperature and solar radiation vary little during the growing season for any particular site in Nicaragua; it is rainfall that has the greatest climatic influence on drybean production. The optimum rainfall is between 300 and 400 mm while Jaramillo [46] quoted by Rios and Quiros [47] found that the maximum yields were obtained with 400 mm precipitation distributed according to the water requirements of the crop.

### 2. Methodology

**Figure 1:**
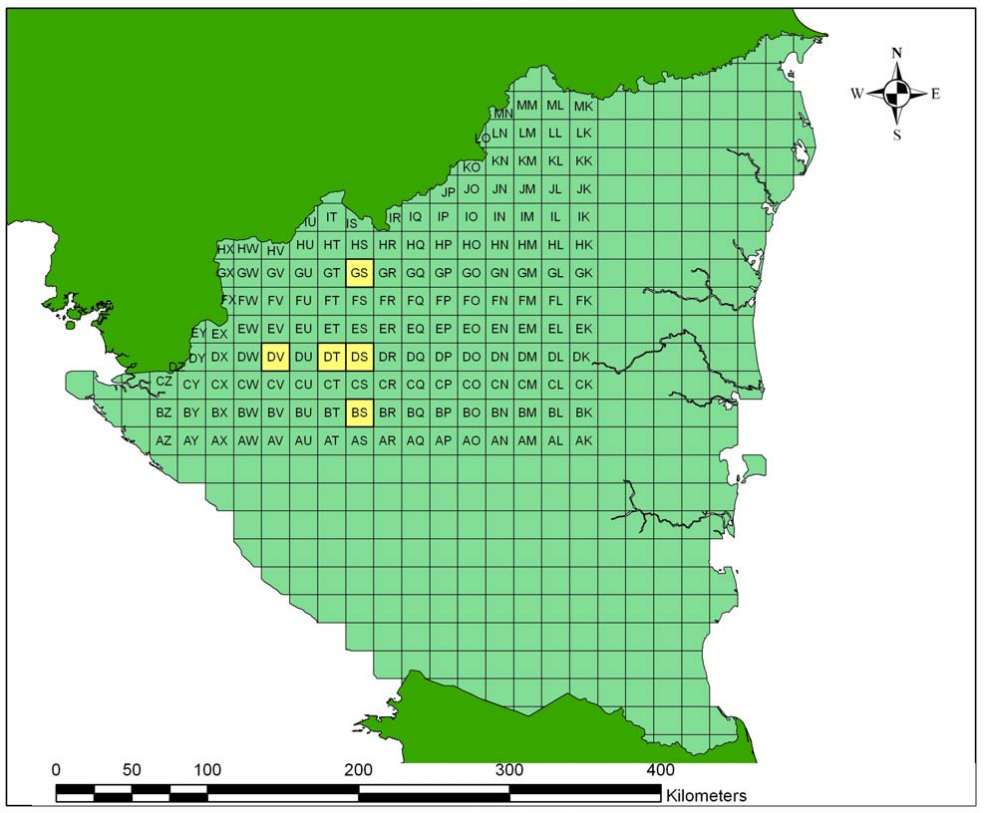
Two letter codes of each pixel used to identify the generated weather data.

We selected the 151 10-arc minute pixels that covered the departments of Matagalpa, Jinotega, Estelí and Nueva Segorvia where drybeans are grown (Figure 1). We generated 99 years of weather data in MarkSim using the coordinates of the geographical center of each pixel. For each pixel, we input these data into the Decision Support System for Agrotechnology Transfer [40] drybean model to simulate yields for the 99 years for eight generic soils with textures ranging from sand to silty clay and either deep or shallow profile from the DSSAT soil database. We used the genetic coefficients for the variety Rabia de Gato, whose physiological characteristics are similar to the traditional varieties grown in the region. In total we simulated almost 120,000 separate crops of drybeans.

For each soil within each pixel (called a “run”), we established the minimum water requirement (MWR, as rainfall) for each dekad below which there was a yield reduction, We tabulated the rainfall data for each dekad with the simulated yield and for each dekad we estimated plausible values for the minimum MWR. We subtracted these MWRs from the observed rainfall for each dekad to calculate deficits, that is, we ignored positive values. The total rainfall deficit for the growing period was therefore the sum of all the deficits. Note that the MWR is a simply a plausible starting value, which is subsequently adjusted in the optimization procedure in the next step.

We selected the lowest quartile of each run and calculated total rainfall deficits from day −10 to day +70 for each simulation within this subset. We then calculated the regression coefficient of total deficit on crop yield. We optimized the estimates of MWR for each dekad to maximize the correlation coefficient using the Solver procedure of Excel with the constraint that MWR for each dekad ≥0. The upper and middle quartiles of yield have rainfall deficits of zero, and therefore were not relevant to establish MWRs. We then calculated the rainfall index for each run as the sum of the MWRs.

The procedure for the deep loam for the pixel BS (Figure 1), which contains the locality of San Dionisio (12° 45′, 85° 51′W), is summarized in Table 3.

**Table 2:** Summary of main challenges that need to be addressed and possible areas of action.

Basis risk	Details	Solutions
Temporal risk	The level of impact of a weather phenomenon will vary according to thetime at which it occurs during the crop cycle. E.g. a shortage of rainfallat just before maturity may kill a crop, whereas just after seeding may havelittle effect.	Indices that represent the temporal variability in sensitivity to rainfall deficit.
Spatial risk	A rainfall deficiency may occur at one location causing crop losses, but thisrainfall deficiency did not occur at the recording location and so no paymentis triggered.	Offset the risk by offering site-specific contracts that account for spatial variability.
Crop specific risk	A rainfall deficiency may kill a drought sensitive crop, whereas a droughtresistant crop will survive through longer periods of drought.	Offset the risk by tailoring the insurance to specific crops.

(Source: World Bank, 2001).

**Table 3:** Sample insurance contract.

RAINFALL INSURANCE CONTRACT										
**REFERENCE WEATHER STATION**				*(e.g.)* San Dionisio INETER weather station						
Crop				*(e.g.)* Dry beans – drought tolerant type						
Reference soil type				*(e.g.)* Deep sand						
Sowing window				*(e.g.)* 15 May to 15 June						
Sowing date rule				*(e.g.)* First day after 5 consecutive rainy days over 5 mm each						
Trigger value				*(e.g.)* −70 mm						
Premium price				*(e.g.)* US$3						
Indemnity				*(e.g.)* US$5 for every mm of rainfall deficit after the trigger valu*e*						
**Minimum rainfall requirements (given crop and soil stated above)**										
	**Day 1 to 10**	**Day 11 to 20**	**Day 21 to 30**		**Day 31 to 40**	**Day 41 to 50**	**Day 51 to 60**	**Day 61 to 70**	**Day 71 to 80**	**Day 80 to 90**
MIN	0	10	10		25	40	40	40	30	0
RAIN										
DEF										
	*a*. *TOTAL Rainfall deficit*									
**Calculation of indemnity payments:**										
1. MIN is the minimum rainfall that is required for your crop in each of the 10 day windows.																				
2. RAIN is the rainfall observed at the reference weather stations (you may enter this into the RAIN box, however it is the official rainfall recorded at the weather station that determines whether you are entitled to an indemnity payment).										
3. DEF is the rainfall deficit. This is calculated by subtracting MIN from RAIN (only negative values are taken into account).										
4. Indemnity payments occur when the TOTAL rainfall deficit is equal to or less than the trigger value.										
5. The rainfall deficit is the sum of the 10 day rainfall deficits.										

### 3. Results

We applied the method to each soil-profile depth combination of the 151 pixels, but the rainfall indices for soils differed little so we present means.

**Figure 2:**
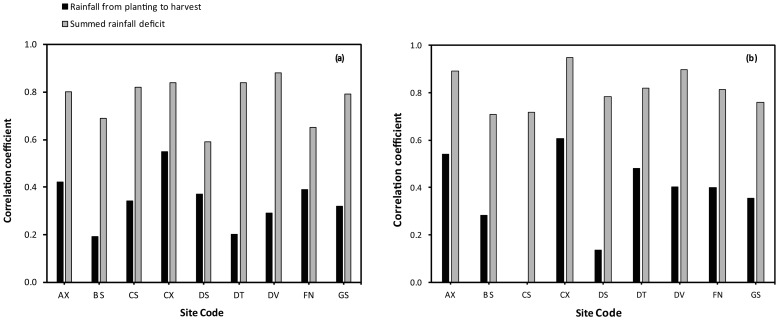
Correlation coefficients of total rainfall deficit and rainfall on yield of drybeans simulated by the DSSAT drybean model on contrasting soils for a selection of sites in north-central Nicaragua. Soil textures are (a) sand, and (b) silty clay. The rainfall for each cell was generated using the MarkSim procedure.

The correlation of the rainfall index with crop yield was in general satisfactory with R^2^ 0.7–0.9 and higher (Figure 2). Soil texture and slope affected the R^2^ values because there is more runoff of rainfall on the heavier soils and particularly on sloping land.

We used a range of generic soils with both deep and shallow profiles. As expected, sandy soils were much droughtier than heavier-textured soils and especially if they were shallow.

**Figure 3:**
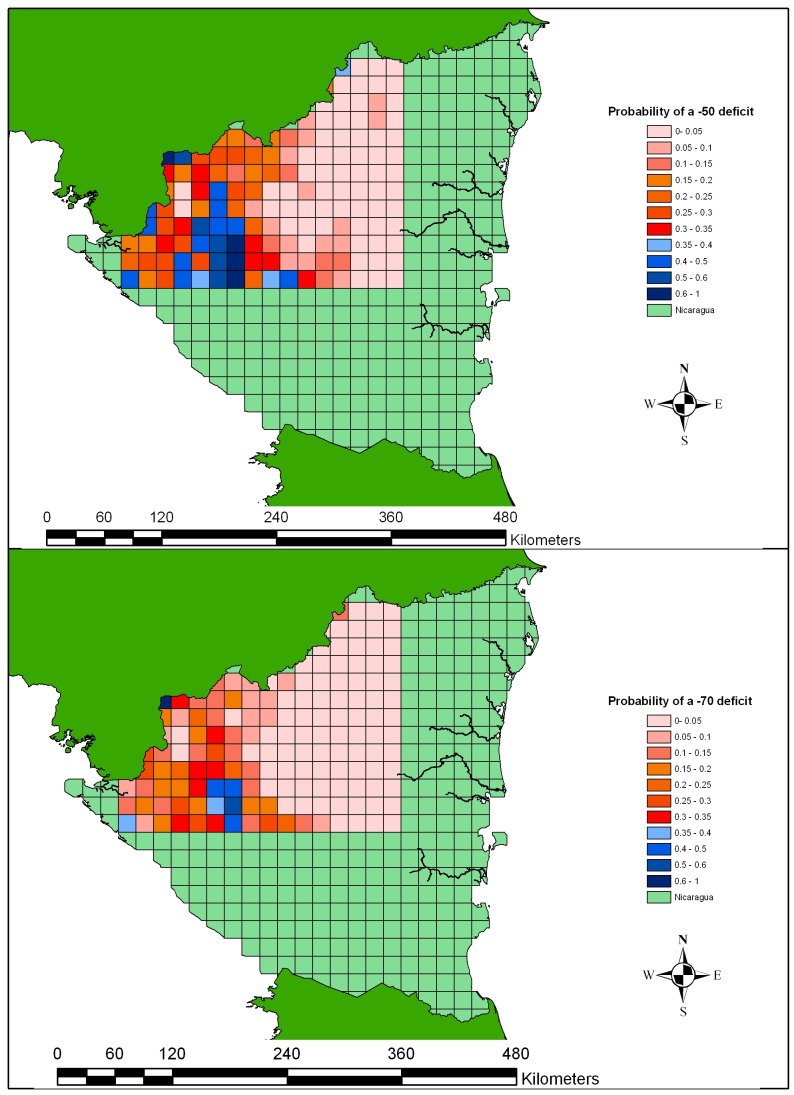
Probability of accumulated rainfall deficits of 50 and 70 mm during the growth of dry beans during the first growing season in north central Nicaragua.

Using the relation of total rainfall deficit against yield we set levels of deficit that would trigger an indemnity payout in a hypothetical insurance instrument. The probabilities of reaching a given level of deficit were then calculated for each of the eight soils for each pixel. The probabilities of reaching deficits of 50 and 70 mm, averaged over all eight soils for simplicity, are presented in Figure 3.

Based on these data, it was then straightforward to design an insurance instrument for each soil within each pixel. The details of a hypothetical contract are shown in Table 3. Tables 4 and 5 show hypothetical growing seasons that do not reach, and do reach, respectively, the trigger level. In designing an insurance instrument based on modeling as described above, it is relatively simple matter to obtain the information necessary for the actual soils in question and adjust the index criteria accordingly.

**Table 4:** Example of a season not entitled to an indemnity payment (total rainfall deficit does not reach the trigger value of −70 mm).

	Day 1 to 10	Day 11 to 20	Day 21 to 30	Day 31 to 40	Day 41 to 50	Day 51 to 60	Day 61 to 70	Day 71 to 80	Day 80 to 90
**MIN**	0	10	10	25	40	40	40	30	0
RAIN	34.9	22.4	0.6	33.8	0	57.6	73.4	161.8	112.9
DEF			*−9.4*		*−40*				
	*a. TOTAL Rainfall deficit*								*−49.4*

**Table 5:** Example of season resulting in an indemnity payment (total rainfall deficit exceeds the trigger value of –70mm).

	Day 1 to 10	Day 11 to 20	Day 21 to 30	Day 31 to 40	Day 41 to 50	Day 51 to 60	Day 61 to 70	Day 71 to 80	Day 80 to 90
**MIN**	0	10	10	25	40	40	40	30	0
RAIN	5.8	3.6	0	9.5	4.1	23.5	12.6	2	96.1
DEF		*−6.4*	*−10*	*−15.5*	*−35.9*	*−16.5*	*−27.4*	*−28*	
	*a. TOTAL Rainfall deficit*								*−139.7*

This exercise shows that it is feasible for any given location to simulate the yield of any particular crop for which there is a simulation model in the DSSAT series.

## Discussion

Sound insurance requires best estimates of hazard probability. It also requires agreement about the likelihood of the hazard occurring. Errors in estimation of the hazard can be due to three sources:

- An incomplete model in which the weather event cannot be related to the loss,
- (a) Spatial and (b) temporal variation in which the model is complete, but data are incomplete, and
- Basis risk.

We discuss each of these as they apply to the Nicaragua case study.

### 1. Incomplete Model. Exclusion of Major Factor Such as Soil and Crop Cultivar

#### 1.1 Soil specificity

The effectiveness of rainfall is strongly influenced by soil characteristics. In soils that have low water-storage capacity, the impact of rainfall shortages will be felt much sooner than in the case of soils with high water-storage capacity. Conversely, when soils are dry, small falls of rain can be more effective on sandy soils compared with clay soils, which require more water to “wet up”. Soil texture, soil depth and water-holding capacity are key factors to take into account in designing an effective insurance scheme. Farmers growing crops on very risky soils will need indemnity payments more often than farmers on less risky soils, which must be reflected in both a soil-specific payout structure and in the cost of the insurance coverage.

#### 1.2 Cultivar specificity

Rainfall requirements will also vary greatly from crop to crop and within the same crop depending on the cultivar. Drought-tolerant varieties will naturally withstand rainfall deficits more successfully than drought-sensitive varieties. Therefore in order to improve the relationship between the rainfall weather index and crop losses, the rainfall indices need to be tailored specifically to the crop variety.

The implications of this for modeling are that the genetic coefficients must be known for the cultivar or cultivars in question. Ideally these should be the outcome of carefully-designed experiments. Nevertheless, it is possible to make some informed guesses as to what the coefficients should be, based on phenological data from different latitudes for the cultivar in question. But the guessing should only be undertaken by experts with a clear understanding of how the particular model represents physiological factors such as photoperiod response and the thermoregulation of plant development.

#### 1. 3 Planting date

In rain-fed agriculture, which is implicit in designing a drought index, sowing date varies from season to season depending on the onset of rain at the start of the growing season. Since weather insurance schemes will be sold in advance when there is no information about what the weather will be, a transparent system is needed that incorporates variable planting dates into the insurance products. Both insurer and insured will need to know the exact start and end dates within which the observed rainfall will be taken into account for determining indemnity payments. To maximize the effectiveness of the insurance product, the method used to establish the sowing date used in the product must reflect the actual planting date as closely as possible.

### 2. Spatial Error

Crop yields from research stations are typically 30%, or more, higher than those of farmers’ fields [48], so that using them as the basis for estimating the effect of a given weather event on farmers’ yields is dangerous. Moreover, weather risk varies spatially. To reflect this spatial variation of risk in the premium, methods to estimate it in risk evaluation are needed so that the insured pays the price of the risk they actually confront.

The spatial limitations of MarkSim’s weather surface, 2.5 arc minutes for Asia (4 km near the Equator), 10 arc minutes elsewhere (18 km), are now irrelevant with the availability of the WorldClim surface [49]. WorldClim’s surface has a resolution of 30 arc seconds, or about 1 km at the Equator and it is a simple procedure to extract data from it and use these as external input to MarkSim. This permits further lessening of basis risk, in all but extreme terrain, where it is unlikely that insurance would be considered.

### 3. Temporal Error, Estimating Extreme Events from Short-run Data

It is common to think that 50 years’ (or so) weather data is sufficient to estimate yield variation in crops. We caution that this is a dangerous assumption. Engineers design structures and other works to withstand a given frequency of extreme weather, for example, a river levy to withstand a one in 100 year flood, termed more simply a 100-year flood. Clearly, a short run of historical data (50 years or even less) is only a limited sample of a very large population. Using such limited data alone to generate probabilities of climate risk will lead to seriously under- or over-estimated risk since by definition, only the extremes encompassed by the actual data are represented.

A different component of temporal factors is some method of incorporating the El Niño-Southern Oscillation (ENSO) phenomenon. Recent studies have shown that the ENSO has a profound effect on weather, not only in the eastern Pacific but more generally globally. Although this may make long-term forecasts more reliable, it is not yet clear how this can be applied in practical terms. MarkSim does not attempt to identify the ENSO phenomenon, although it does include its effect in the temporal variation it represents.

No weather simulator will forecast extreme events, so the method presented here will need to be modified to take account of their historical frequency if that is deemed necessary [35]. As it stands, the method does not address this issue. Typically, engineers use a Pearson function (logarithmic extrapolation) based on historical data, but consideration of this approach is outside the scope of this paper.

### 4. Consequences of Basis Risk

As the checkered history of insurance shows, commercial viability is essential to ensure a self-sustaining insurance process. Viability of insurance is determined by the design of the insurance process, which encourages risk-sharing on the basis of transparent agreements between the insurer and the insured about drought probabilities. A key part of this agreement is the provision of accurate estimates, and in this respect we have concerns about potentially imprudent application of insurance. Insurance with excessive basis risk will be expensive or, worse, may invoke moral hazard since farmers will believe themselves to be protected whereas in fact they are not.

Index-based schemes seem particularly vulnerable to basis risk, since their prime attraction is cost reduction through insuring weather events rather than actual inspectable loss. We discuss this point in more detail in Diaz Nieto [12], but briefly the actuarial component of the index instrument is calculated on the basis of the data recorded at a particular meteorological station, which is also where the current rainfall is measured on which a payout will be assessed. Any gradient in the actual climate surface from the station to a farmer’s fields constitutes basis risk, *which is borne by the farmer*. Proponents of indexed insurance and assessments of unsuccessful pilot schemes consistently ignore this, in the latter case often expressing bewilderment that farmers are unwilling to avail themselves of the offered instrument. We have seen no case where the reasons for the farmers’ unwillingness to buy have been disaggregated to include farmers’ perception of the suitability of the instrument for *their own farms*.

We believe that basis risk is a key issue and minimizing it is a major advantage of the scheme we propose here. There can be as many insurance instruments as are necessary to provide coverage that individual groups of farmers perceive to be relevant to them as the procedures we describe here can generate pseudo-historical data of both weather and crop yield for any point for which they are needed. The only requirement is that each will be required to have its own rain gauge on which to determine any payout. In a successful scheme for maize farmers in western Kenya in which we had some involvement, cell-phone masts were the sites of choice for the rain gauges, with the data being recorded in near-real time, astonishingly, in Austria, from where it was readily available to both the insurer and the insured.

### 5. Practical Implications: Technical Considerations in the Design of an Effective Weather Insurance Scheme

A weather-index insurance scheme should ideally take into account the following scientific and technical details:

#### 5.1 Payable index

Several models, typified by the DSSAT series, are available to simulate crop yield. The minimum climatic variables required as key drivers are daily maximum and minimum temperatures, solar radiation and rainfall. In principle, such models could be used to determine whether farmers receive an indemnity or not, by inputting the current weather data into the model as they become available. Although this approach is scientifically sound, it is unlikely to be thought transparent by either the insured or the insurer. The requirement of a weather index simply means that a complex relationship between one climatic variable, such as rainfall in the case of drought, and crop yield must be converted into a simple index. Moreover, the index must be easily understood by all parties so that the trigger event for an indemnity payment and its magnitude is clearly defined.

#### 5.2 Accurate estimation of payment probabilities

Insurance companies will need to know how often they will be paying out indemnities based on each of the weather stations they are using as a reference for payments. In some cases these weather stations will not have the necessary historical data to determine this probability. A method therefore needs to be established that will enable accurate estimation of the probability at points where the historical data are inadequate or lacking.

#### 5.3 Weather insurance package or stand alone solution crop solution

The kind of weather index method presented in this paper is applicable to any crop included in DSSAT, which are the main staple crops grown. Furthermore, the index developed for any one component may be part of a broader insurance package, which includes, for example, excessive precipitation and other risks, or as a stand-alone solution. The type of instrument offered will depend on the geographical location and the risks that farmers there face.

Of course, the approach that we propose must be validated in the real world, and we present it as a paradigm that can address the needs of smallholders who lie outside the ambit of current schemes. These validations can only be done in the future (unless there is some trove of data of which we are not aware), with a well-distributed set of weather stations and reliable data of farmers’ yields in the vicinity of each. A scheme in process by the Syngenta Foundation in Kenya collects weather data in real time from sensors on cellphone masts for farmers located in the coverage area of the tower. Yield data from nearby farmers’ fields could provide the validation that we seek over several years.

#### 5.4 Methodological issues

We discussed the suitability of the DSSAT models to formulate indices for crop weather insurance in Diaz Nieto et al. [12], especially the criticism that ‘DSSAT results are calibrated to a very specific and idiosyncratic situation’ [8]. We disagreed with this assessment, pointing out that, ‘far from a weakness, this is [DSSAT’s] great strength’. We argued that DSSAT allowed us to reduce spatial risk in a ‘transparent and logically consistent manner …, which is impossible in the statistical approaches advocated by others.’ We went on to cast doubt on the DSSAT modeling that Osgood et al. [8] did, concluding that, ‘In our experience, the results that Osgood et al. [8] report are so bad that we wonder whether the simulations were set up correctly. Certainly DSSAT can give bad results if the models are not set up with some basic understanding of crop agronomy.’

We note that Gianini *et al*. [9], in examining artificially-generated weather for sites in Central America, used the WGEN routine, which uses a first-order Markov model. As Jones and Thornton [50] point out, first-order Markov simulates temperate weather, which is controlled by a more-or-less orderly procession of weather systems from west to east, relatively well, but it fails to capture the very different synoptic situations of the tropics. Jones and Thornton [50] showed that a third-order Markov model was required to simulate the different patterns of rainfall in the tropics, producing MarkSim [39], which we have used here. We urge others to make use of this tool that more closely reflects the behaviour of tropical systems.

No weather simulator will forecast extreme events, so the method presented here will need to be modified to take account of their historical frequency if that is deemed necessary [35]. As it stands, the method does not address this issue. Typically, engineers use a Pearson function (logarithmic extrapolation) based on historical data, but consideration of this approach is outside the scope of this paper.

The task of producing index insurance instruments for smallholders anywhere in the tropics is frustrated by two realities: there are few long-term sets of meteorological data (and let us not even think about how reliable they might be), and data of farmers’ yields are similarly sparse and unreliable. Rather than treat the problem as intractable and ignore the needs of smallholders that conventional approaches regard as uninsurable, which most insurers and especially reinsurers do, we propose an alternative. MarkSim does reliably represent the climate variability in the tropics, especially when combined with the WorldClim database at 1-km resolution. The DSSAT suite of crop models incorporates understanding of crop physiology, biochemistry, and agronomy, developed over more than 30 years and have been widely documented in the literature over the last 30 years. Thus we feel that it is realistic to combine modeled weather data extrapolated from the best available meteorological data in combination with a tested crop model to generate reasonable predictions of risk in areas that are currently unserved by commercial approaches.

### Conclusion

We present methods of providing low-cost, site-specific drought insurance products for most crops in any location in the tropics. We explain the benefit of insurance to risk takers, and especially those with minimal resources, from which it should become apparent that the major contribution this innovation offers is that it streams best available science about natural hazards directly to decision makers, through the medium of commercially-viable insurance products. Insurance provides decision-support to manage drought risk. The basis of the method, the insurance premium, transmits the best-available estimate of drought probabilities.

Estimates are only as accurate as the predictive model that produces them and we reflect here on three sources of basis risk that are likely to occur when modeling crop drought risk: structural uncertainty of the model; spatial error and temporal error. Structural uncertainty increases when the model fails to represent processes that significantly influence drought risk. In this respect, a model that depends solely on correlation between rainfall and yield will not represent systematic and significant yield variations that are caused by temperature, soil, crop variety or a number of other factors.

Spatial error introduces a second major source of basis risk, since it is rare that weather data, and even more so, yield data, are available with sufficient density to enable simple interpolation over large areas. Even where dense networks of weather stations exist the degree of bias towards non-marginal sites is unknown, hence its ability to represent higher risk in marginal areas. Thirdly, error can occur due to unexplained temporal error caused by inadequate data runs. A purely empirical estimation of low probability events requires long runs of data.

We do caution that the method should not be applied uncritically as illustrated by the effect of soil texture and slope on soil water recharge, and the influence of temperature on growth and hence yield at higher altitudes.

## Methods

### 1. The Main Challenge in Developing Weather Insurance: Basis Risk

The greatest challenge facing weather-based insurance products is basis risk [14], [26], [32], [37], [38]. Basis risk occurs when the insurance index does not accurately represent loss: a weather index may not trigger a payment when there has indeed been a loss; or payment may occur without serious loss. The insurance product will not be attractive to potential customers if they think that the basis risk is too high [26].

A feasibility study of rainfall indices for Nicaragua concluded that even within departments a single index did not adequately represent the spatial variability of risk [14]. In each department there was at least one weather station where the data were markedly different from the others. A study by Diaz-Nieto *et al*. [12] using simulated data for Honduras also revealed that a single weather index was not appropriate for a country the size of Honduras.

Basis risk is caused by the need to model complex heterogeneous systems within a single index. There are three sources of basis risk (Table 2).

Specialized contracts can be designed to offset much of temporal, spatial and crop-specific basis risk [37]. However, doing so may increase administrative costs and, more importantly, increase the complexity involved in marketing and distribution. An alternative to overcome basis risk is a larger number of standard contracts that cover all possibilities and priced accordingly, and allow the insured to select the contract they consider most appropriate [38].

### 2. Establishing the Correlation between Crop Yield and the Rainfall Index

The fundamental requirement of a rainfall index is that rainfall must explain a large proportion of the variability in yield [26], [30], [32], [38]. As a first step, it is essential to establish the cause and effect relationship [38], so that the index represents critical rainfall deficits that account for crop yield losses. It is not sufficient, for example, to posit that a rainfall deficit of 30% of the long-term average will trigger payment because this provides no information about the timing of rainfall in relation to crop demands at different growth stages.

Defining the weather events that cause the most serious yield losses and that cover as many of the loss-causing events as possible requires a considerable investment in research [26]. Furthermore it is critically important that both parties agree that the weather index adequately explains the variability in crop yields [30]. Few customers would be inclined to purchase insurance that they did believe protected them against risk.

### 3. Limited Availability of Yield and Climate Data on which to Base Indices

Stoppa and Hess [30] suggested that to develop effective weather-index insurance the weather variable must not only be measurable but adequate historical weather records must be available from which to estimate probabilities of a risk event occurring and its magnitude. In spite of this, many of the feasibility studies into the use of weather-based indices in developing countries provide indices based on relatively few data. Reliable long-term datasets of weather in developing countries are very limited and this presents a major potential challenge. It is noteworthy that countries with poor infrastructure are amongst those places where an effective insurance product could have most impact. The danger is that poor regions, which have greatest need for insurance, are those which are excluded, precisely for reasons of poor infrastructure associated with poverty.

An alternative approach, which we describe below, is to use statistical models and process-based simulation models, based on decades of scientific analysis, to generate ‘pseudo-historical’ data of climate and yield. Where possible these pseudo-historical data can be complemented with such weather data as are available.

### 4. Payout Index Highly Correlated with Yield Loss

In a weather insurance scheme it is not the actual crop loss that is insured but the loss-causing event, which in this case is a specified adverse weather event. Therefore the way in which the relationship between weather and crop losses is expressed in an insurance index needs to be carefully thought out and appropriately designed. A producer will be interested in a weather-insurance scheme that is highly likely to pay out when (s)he does indeed suffer a crop loss. Ideally the relationship between weather and crop yield can be extracted from long historical records of both. In practice, as in the case of drybean yields in Nicaragua, data are typically very scarce. It was therefore necessary to design a methodology that allowed weather insurance to be developed in these circumstances.

### 5. Summary of the Honduras Study

Díaz Nieto *et al*. [12] proposed a method for an indexed insurance instrument for tropical sites for which historic data were not available for either rainfall or crop yield. Briefly, they combined the MarkSim weather generator [39] with the drybean simulation of the DSSAT series of crop models [40] for six sites in central Honduras. Because there was a low frequency of drought for some of the sites, they randomly imposed droughts for ten-day periods (dekads) during crop growth. By comparing the simulated yields of the droughted crops with those with no drought, they determined sensitivity coefficients of the crop to drought at different stages of growth for each site. Rainfall for each dekad of crop growth was weighted by the crop sensitivity coefficient and the total weighted rainfall was expressed as a percentage of the long term mean. They selected an arbitrary “strike” value for each site for years with rainfall deficit greater than 65%. They based payout on the percentage deficit and calculated notional premiums based on the frequency and amount of payout.

Because the north-central mountains of Nicaragua are drier than the six sites in Honduras chosen by Díaz Nieto *et al*. [12], we used a different method to determine the crop sensitivity coefficients, described below.
